# Relevant human tissue resources and laboratory models for use in endometriosis research

**DOI:** 10.1111/aogs.13119

**Published:** 2017-04-05

**Authors:** Erin Greaves, Hilary O.D. Critchley, Andrew W. Horne, Philippa T.K. Saunders

**Affiliations:** ^1^ MRC Centre for Reproductive HealthThe University of EdinburghQueen's Medical Research InstituteEdinburghUK; ^2^ MRC Centre for Inflammation ResearchThe University of EdinburghQueen's Medical Research InstituteEdinburghUK

**Keywords:** Endometriosis, in vitro, in vivo, ex vivo, laboratory models

## Abstract

Endometriosis is characterized by the growth of endometrium‐like tissue outside the uterus, most commonly on the pelvic peritoneum and ovaries. Although it may be asymptomatic in some women, in others it can cause debilitating pain, infertility or other symptoms including fatigue. Current research is directed both at understanding the complex etiology and pathophysiology of the disorder and at the development of new nonsurgical approaches to therapy that lack the unwanted side effects of current medical management. Tools for endometriosis research fall into two broad categories; patient‐derived tissues, and fluids (and cells isolated from these sources) or models based on the use of cells or animals. In this review, we discuss the literature that has reported data from the use of these tools in endometriosis research and we highlight the strengths and weaknesses of each. Although many different models are reported in the literature, hypothesis‐driven research will only be facilitated with careful experimental design and selection of the most appropriate human tissue from patients with and without endometriosis and combinations of physiologically relevant in vitro and in vivo laboratory models.

**Abbreviations**
c‐jun: V‐jun avian sarcoma virus 17 oncogene homolog
DRG: dorsal root ganglia
EP2: prostaglandin E2 receptor
EPHect: Endometriosis Phenome and Biobanking Hormonization Project
ER: estrogen receptor
GWAS: genome‐wide association studies
HEECs: human endometrial endothelial cells
hTERT: human telemorase reverse transcriptase
HUVECs: human umbilical vein endothelial cells
*K‐ras*: V‐Ki‐ras2 Kirsten rat sarcoma viral oncogene homolog
L‐THP: levo‐tetrahydropalmatine
NK: natural killer
Prkdc^SCI^: loss‐of‐function mutation in the mouse homologue of the human *PRKDC* (severe immunodeficient mice)
TGF‐β_1_: transforming growth factor β_1_
TIMP‐1: tissue inhibitor of matrix metalloproteinase 1
WERF: World Endometriosis Research Foundation

**Key Message**

Due to its complex etiology and pathophysiology, endometriosis research requires careful selection of appropriate in vitro and in vivo models that, in combination with the use of well‐characterized human tissue can enhance the identification of anxiously awaited new therapies.

## Introduction

**Figure 1:**
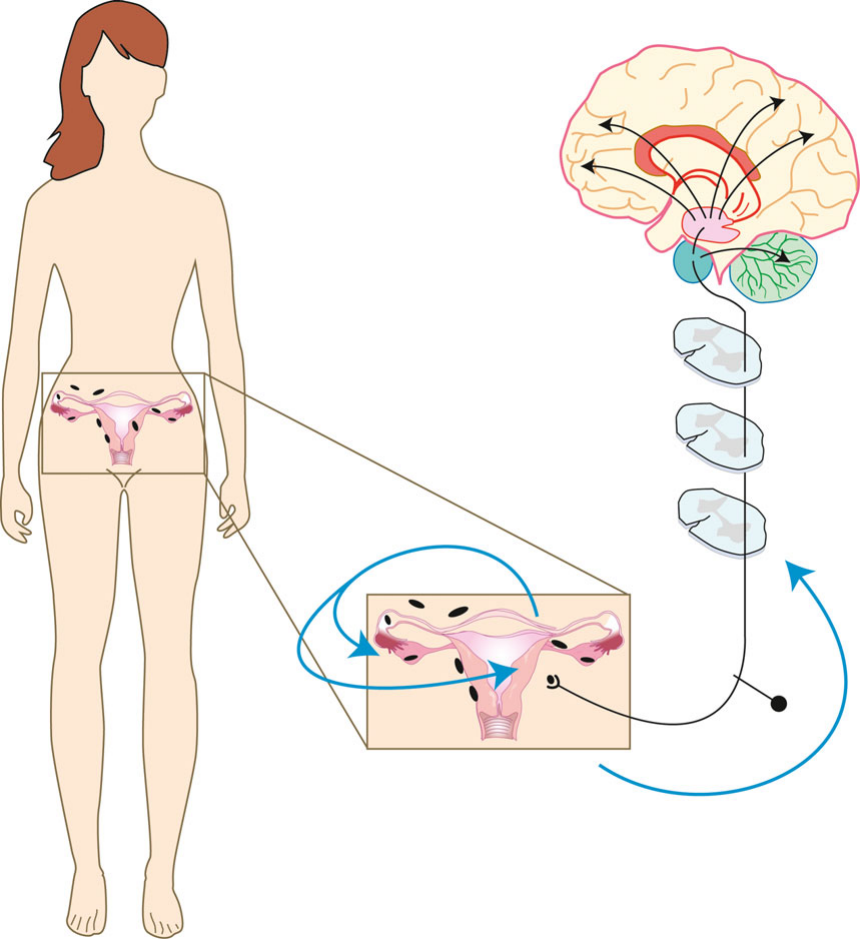
The presence of endometriosis lesions impacts the reproductive system and the nervous system. Endometriosis lesions present within the pelvic cavity cause the defining symptoms of the condition, which are infertility and chronic debilitating pelvic pain. Lesions generate an inflammatory environment, which may have a negative impact on developing oocytes and implanting blastocysts. This inflammation is also thought to activate nerve fibers that innervate lesions. In addition to disease‐specific cellular and molecular changes, extensive lesions and adhesions can cause distortion of the pelvic organs and nerve compression that may also contribute to fertility problems and pain. Animal models of endometriosis are required to dissect out specific disease mechanisms that impact on the reproductive and nervous system. [Color figure can be viewed at wileyonlinelibrary.com]

Endometriosis is characterized by the growth of endometrium‐like tissue outside the uterus, most commonly on the pelvic peritoneum and ovaries 1. The presence of ectopic endometrial deposits (lesions) in the peritoneal cavity are thought to cause the defining symptoms of endometriosis, which are debilitating pelvic pain and infertility. Hence, these multicellular tissue deposits impact at least two biological systems; the nervous system and the reproductive system (Figure 1).

**Figure 2:**
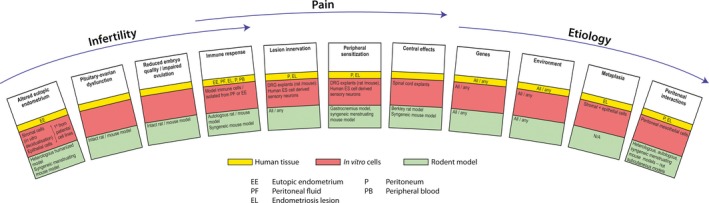
Research question specific models for the study of endometriosis. Many theories on the pathophysiology and etiology of endometriosis exist. Hypothesis‐driven research will be facilitated with careful experimental design and selection of appropriate human tissue from patients with and without endometriosis and appropriate combinations of in vitro and in vivo laboratory models. [Color figure can be viewed at wileyonlinelibrary.com]

To identify new means of treating the symptoms of endometriosis, it is critical to understand the complex pathophysiology of the condition. However, not only are there multiple theories on the origin of endometriosis (retrograde menstruation/transplantation, metaplasia), there are many different proposed theories on its pathogenesis (such as genetics, immune response, environment). Hence, there are multiple considerations and end points to consider during the design of experiments aimed at identifying potential therapeutic targets for endometriosis. Tools for endometriosis research fall into two broad categories: patient‐derived tissue and fluid and cells isolated from these sources, or models (in vitro and in vivo; Figure 2). Each have pros and cons and have differing utilities depending on the research question. In the current review, we discuss relevant human tissue resources and laboratory models for use in endometriosis research and we highlight the strengths and weaknesses of each.

## Material and methods

We conducted a primary computerized literature search for relevant publications in “PubMed” that related to laboratory models for identifying therapeutic targets for endometriosis. We searched using the following key words: endometriosis AND human tissue OR mouse model OR rat model OR animal model OR in vitro. One author (EG) selected relevant abstracts and the full texts were obtained. EG reviewed studies that were published from 2000 to 2016 and although those studies formed the basis of our review, some older publications were included as deemed appropriate for historical but pivotal models of endometriosis. Reference lists from other publications were also examined for any relevant studies that were not extracted from the initial literature search. Articles were included if they (i) described the use of human tissue for the identification of novel genetic traits or pathways that could represent therapeutic targets for endometriosis (studies using human tissue were included only if patients and controls were confirmed via laparoscopy to have or not have endometriosis), (ii) described a unique in vivo rodent model of endometriosis, or (iii) described relevant in vitro techniques for the study of endometriosis. This is not a systematic review.

## Patient‐derived tissue, fluid and cells

### The appropriate methodology for the collection of patient‐derived material

The analysis of human samples has provided valuable advances in our understanding of biological changes associated with endometriosis in both the peritoneum and the endometrium. As yet there are no validated clinical biomarkers of endometriosis 2, so the reference standard for diagnosis of the condition is the macroscopic visualization of lesions during laparoscopy. The procedure provides clinicians with an opportunity for the collection of tissue and fluid biospecimens for use in endometriosis research.

Many centers worldwide have been collecting tissue and fluids from patients with and without endometriosis undergoing laparoscopy, as well as surgical and clinical phenotype data for a range of research purposes. However, huge variations in the collection of such data and specimens exist that could limit comparisons in data and reproducibility of results from different studies. The World Endometriosis Research Foundation (WERF) Endometriosis Phenome and Biobanking Harmonization Project (EPHect) spearheaded a mission to promote the adoption of internationally agreed standard operating procedures for tissue and fluid sample collection, processing and storage as well as surgical and clinical phenotype data collection. The WERF EPHect working group developed surgical and clinical questionnaires and evidence‐based standard operating procedures that were published in four concurrent articles 3, 4, 5, 6. This standardization will optimize sample quality, reduce variability and enable large‐scale cross‐center, epidemiologically robust endometriosis research. At the time of writing this review there are 13 registered centers using the WERF EPHect tools (http://endometriosisfoundation.org/ephect/centres-using-werf-ephect-tools/). A list of human biological specimens being used in endometriosis research is provided in Table 1.

**Table 1:** Human tissues and fluid biospecimens used in endometriosis research

Tissue	Fluid
Eutopic endometrium	Blood
Ectopic endometrium (lesion)	Urine
Unaffected peritoneum (adjacent and distal or prone and distal)	Saliva
Myometrium	Peritoneal fluid
Subcutaneous abdominal fat	Endometrial fluid
Omental/visceral fat	Menstrual effluent

Lesions vary in their location and invasiveness, biopsies can therefore be from the ovaries (endometriomas), the peritoneal lining, or deep infiltrating nodules. This heterogeneous nature of lesion biopsies, presentation of the condition, disease classification, variation in sample metadata, and the lack of correlation between perceived severity of disease and symptomatology can make data generated in endometriosis studies difficult to interpret. Standardization of clinical phenotype data collection and biopsy recovery through WERF EPHect will enable the generation of more reliable and comparable data. Samples should also be explicitly characterized and comprehensive details of sample metadata (cycle stage, disease severity, pain scores, history of subfertility) should be published alongside any results using human tissue to ensure transparency of any potential confounding factors.

**Figure 3:**
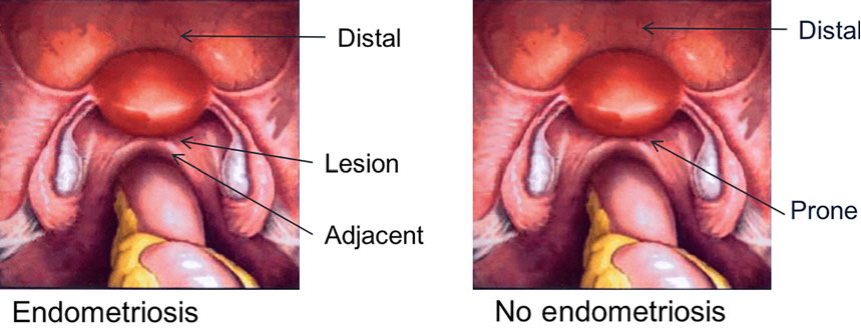
Peritoneal and endometriosis lesions collected at time of surgery. Taking biopsies of peritoneum from sites adjacent and distal to lesions in patients with endometriosis, and from sites prone to endometriosis in patients without the condition provides useful biological information when analyzed as additional controls within an experiment. [Color figure can be viewed at wileyonlinelibrary.com]

Appropriate use of experimental controls is critical for human tissue data to be informative. Peritoneum and endometrium control samples should be included in studies that analyze gene expression in endometriosis lesions (particularly homogenized samples), because lesion biopsies are often contaminated with surrounding peritoneal tissue and in these cases it should be included as a control. It is not sufficient to compare gene expression in endometriosis lesions only to the endometrium or peritoneum alone. It is not entirely necessary to include peritoneal controls in immunohistochemical analysis of particular cell types, for example, because it is easy to distinguish the lesion boundary from surrounding peritoneum. Biopsies of peritoneum from sites adjacent and distal to lesions in patients with endometriosis and from sites prone to endometriosis in patients without the condition (Figure 3) also provides useful biological information when analyzed as additional controls within an experiment. Menstrual cycle phase is also known to have obvious impacts on peritoneal fluid concentration 7 and composition, and a profound impact on endometrial 8, and likely also endometriosis lesion, gene expression. Exogenous hormones can also modulate gene expression in these samples. For example, oral contraceptive use increases the expression of cyclooxygenase‐2 in the eutopic and ectopic endometrium of women with endometriosis 9. Stromal cells isolated from ovarian endometriomas exhibit increased expression of aromatase, estradiol 17β‐dehydrogenase 1, steroid sulfatase and estrogen sulfotransferase 10. Whether these gene expression changes are maintained when cells are isolated and cultured is uncertain because the majority of published studies use cells derived from patients that have not had exogenous hormone treatment 3–6 months before laparoscopy. Access to true control samples collected from fertile patients with no pain and no endometriosis is often limited due to the fact that few women undergo laparoscopy (or endometrial biopsy) without symptoms (decline in laparoscopic sterilization). As women may exhibit no symptoms even though they have lesions, consistent with endometriosis detected at laparoscopy, caution is necessary in defining “control” samples.

Below we discuss a limited number of key studies that have significantly enhanced our understanding of endometriosis and also provide examples of identification of genes or pathways that could be targets for therapeutic intervention.

### Identification of genes associated with endometriosis, gene expression trends, and novel pathways

Microarray transcriptomic studies in combination with pathway analysis have yielded informative results on biological changes taking place in the eutopic endometrium of women with endometriosis; Burney et al. demonstrated a dysregulation of the proliferative to secretory transition in women with endometriosis. In secretory phase endometrium of women with endometriosis, the authors identified persistent expression of genes associated with DNA synthesis and cellular mitosis and decreased expression of progesterone‐regulated genes, suggestive of a “progesterone‐resistant” phenotype 11.

Genome‐wide association studies (GWAS) performed on DNA extracted from blood or saliva, in combination with replication studies, have been hugely informative in the identification of genetic loci associated with endometriosis risk and associations with other genetic traits 12, 13. GWAS studies provide an opportunity for identifying new drug targets for endometriosis; genes discovered using this approach can be investigated for their ability to be targeted by small molecule inhibitors and therapeutic antibodies or protein therapeutics. For example, 155 of 991 genes implicated in disease from GWAS (15.6%) have an associated drug project in development 12. The implementation of WERF EPHect standard operating procedures for the collection, processing, and storage of tissue and biofluid specimens will enhance such large‐scale cross‐center collaboration, such as GWAS. Validation of results generated in GWAS studies remains a challenge, as does exploring the mechanistic relevance of identified genes.

Analysis of cytokine profiles of peritoneal fluid samples identified a subset of patients with a shared consensus signature of elevated cytokines associated with macrophage infiltration and activation. Bioinformatics analysis identified an enrichment of V‐jun avian sarcoma virus 17 oncogene homolog (c‐jun), FBJ murine osteosarcoma viral oncogene homolog (c‐fos) and activator protein 1 (AP‐1) transcription factor binding sites among the measured consensus cytokine signature. Subsequent inhibition of upstream kinases of c‐jun resulted in an attenuation of cytokine expression by macrophages isolated from peritoneal fluid from women with endometriosis 14, suggestive of a novel therapeutic strategy for limiting inflammatory mechanisms that drive endometriosis.

### How studies on cells derived from patient tissues/fluids have informed our understanding of the pathophysiology of the disorder

In a study from our group, gene expression analysis performed on human endometriosis lesions compared with endometrium and peritoneum revealed an upregulation of the axonal guidance molecule *SLIT3* in lesions 15. In this same study immunofluorescence performed on human endometriosis lesions indicated that endothelial cells lining the blood vessels of lesions express estrogen receptor β (ERβ) and not ERα, and that blood vessels and nerves are found in close proximity in lesions. These findings informed in vitro and in vivo studies exploring ER regulation of Slits in blood vessel–nerve crosstalk in endometriosis 15. In another study from our team, analysis of peritoneal fluid revealed increased levels of transforming growth factor β_1_ (TGF‐β_1_) and lactate in women with endometriosis, and an increase in glycolysis‐related genes 16. This informed investigation of TGF‐β_1_ regulation of glycolysis genes and lactate levels in peritoneal mesothelial cells from women with and without endometriosis and led to the hypothesis that the “Warburg effect” (a high rate of glycolysis and lactic acid fermentation) seen in tumorigenesis is a key contributor to the pathophysiology of endometriosis and may be modulated by TGF‐β 16. These two studies emphasize the power of integrated studies that use a number of different models to explore hypothesis‐driven endometriosis research.

**Figure 4:**
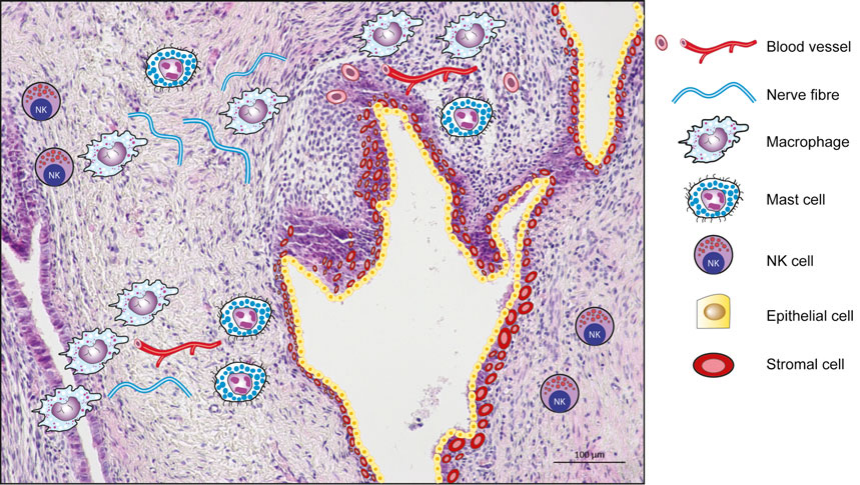
Endometriosis lesions are multicellular tissue deposits. Typical endometriosis lesions contain endometrioid glandular structures (made up of epithelial cells) and stromal cells, similar to the eutopic endometrium. Lesions become vascularized, innervated and infiltrated by immune cells such as macrophages, mast cells and natural killer cells. Isolation of specific cell types from lesions is difficult due to the limited amount of tissue available from biopsies. In most cases the exploration of cellular interactions must be recreated using cell models and disease‐specific effects on physiology must be modeled in vivo. [Color figure can be viewed at wileyonlinelibrary.com]

Endometriosis lesions are complex multicellular tissue deposits (Figure 4). The hallmarks of an endometriosis lesion are the presence of endometrioid epithelial and stromal cells that resemble the cellular organization of the eutopic endometrium. These lesions are highly vascularized and we now know that they are innervated, enabling a dialogue between the lesion microenvironment and the nervous system. Immune cells including macrophages, mast cells, T and B lymphocytes and natural killer (NK) cells are also present within lesions and contribute to the inflammatory microenvironment of the lesion. The pelvic peritoneum may also play an important role in the establishment and progression of endometriosis by providing a surface for the attachment of endometrial fragments. Hence, many different cellular interactions can take place within a lesion and appropriate cells are required for the study of these interactions. To complement studies on intact tissue biopsies a number of studies have also focused on analysis of different cell types isolated from patient biopsies.

#### Eutopic endometrium

Non‐cancerous epithelial cells are difficult to propagate from primary tissue due to their short lifespan and usually enter senescence after 2 weeks in culture. For this reason, only a limited number of studies use epithelial cells from patients with endometriosis. Those that have, indicate altered gene expression and enhanced migratory abilities in epithelial cells isolated from patients with endometriosis 17. Stromal cells are much easier to isolate and propagate from primary tissue and many studies have used stromal cells isolated from the eutopic endometrium from patients with and without endometriosis undergoing laparoscopy. Endometrial stromal cells may be induced to decidualize in vitro using progesterone and cAMP. This approach has been used as a model for studying the potential effects of endometriosis on endometrial receptivity and differences in gene expression in cells from women with and without endometriosis. For example, decreased Notch signaling and connexin 43 expression identified in the endometrium of women with endometriosis have been implicated in impaired decidualization using eutopic stromal cells 18, 19. Immune cells resident in the eutopic endometrium of patients may also be isolated with flow cytometry. For example, uterine NK cells have been analyzed using flow cytometry and found to exhibit decreased levels of killer cell inhibitory receptors 20. Increased levels of uterine NK progenitor cells have also been identified with flow cytometry in the eutopic endometrium and are thought to contribute to endometriosis‐associated infertility 21.

#### Ectopic endometrium

Epithelial cells have been isolated from lesion biopsies and used to explore the effect of inhibition of the Wnt/β‐catenin pathway on gene expression and the functional end points proliferation, migration and invasion 22. The very limited amount of epithelial cells that can be recovered from ectopic endometrium means multiple experiments cannot be performed from cells isolated from one patient and large patient numbers are required for a study. Primary stromal cells isolated from endometriosis lesions have also been used in a number of studies to demonstrate for example, that these cells also have an increased migration and invasion ability 23.

#### Peritoneum

In women with endometriosis the phenotype of the mesothelial cells that line the peritoneal cavity has been reported to be altered, such that ectopic endometrial cells are more likely to attach and invade underlying structures 24. Primary human peritoneal mesothelial cells can be isolated during laparoscopic surgery by gentle brushing of the pelvic mesothelium using a specialized brush, the cells can then be dislodged and established in culture 16. These cell types have been used in studies that shed new light on the role of the mesothelial cell in endometriosis pathophysiology including a Warburg‐like metabolic reprogramming 16, 25, 26, 27. Peritoneal mesothelial cells have also been used in a co‐culture model to simulate interactions between endometrial stromal cells and human peritoneal mesothelial cells in normal and pathophysiological states 28.

#### Immune cells derived from peritoneal fluid or peripheral blood

The peritoneal fluid is an incredibly useful resource for the study of the role played by immune cells in the etiology of endometriosis. T and B lymphocytes, NK cells and macrophages 14 isolated from the peritoneal fluid of women with and without endometriosis have been analyzed in a number of studies 29, 30, 31. The presence of endometriosis has also been hypothesized to effect immune profiles systemically; T, B and NK cells isolated from the peripheral blood from women with endometriosis have also been analyzed 29, 30, 31.

## In vitro and in vivo model systems

### In vitro models

The number of cells that can be isolated from lesions is limiting for many studies. Additionally, nerves for example, cannot be isolated from biopsies and for these reasons cell models are required. Primary patient‐derived cells or cell models provide a limited “snap shot” of gene expression or cell function and at best can be used to provide some information on cell–cell interaction when used in co‐culture or three‐dimensional culture systems.

#### Epithelial and stromal cells

The limited number of cells that can be isolated from endometriosis lesions is often the reason that many researchers decide to use cell lines. Normal human endometrial epithelial cells have been immortalized using human papillomavirus and human telemorase reverse transcriptase (hTERT) 32 and used as a control cell in a number of endometriosis studies. Purified epithelial cells isolated from ovarian endometriomas (E'mosis1 and 2) have also been immortalized by combined transfection of human *cyclinD1*, cyclin‐dependent kinase 4 (*cdk4*) and human telomerase reverse transcriptase (*hTERT*) 33. Another widely used endometriotic epithelial cell line (12Z) was first established from an active peritoneal lesion 34. As each of these cell lines is generated from individual patients, it cannot be assumed that results obtained from their interrogation are a true representation of endometriotic epithelial cells. However, they are useful for the analysis of signaling pathways or functional studies investigating invasion, migration and proliferation that may need more cells than can be isolated from primary tissue. The ectopic stromal cell line 22B (derived from an active red peritoneal lesion) has been used in a number of studies 34, 35 but poses the same limitation as the ectopic epithelial cell line discussed above.

#### Mesothelial cells

The humen pleural cavity mesothelial cell line MeT‐5A has also been used in endometriosis studies and is thought to mirror the phenotype of primary human peritoneal mesothelial cells. A peritoneal mesothelial cell line has been established through transfection with simian vacuolating virus 40 TAg (SV40 T) antigen 36. Peritoneal mesothelial cell line (LP9) is a commercially available peritoneal mesothelial cell line that has been used in a limited number of endometriosis studies 37. Primary mesothelial cells derived from different locations and cell lines are thought to have a similar phenotype to peritoneal mesothelial cells. However, as with most cell lines they are not useful for exploring disease‐specific phenotype.

#### Immune cells

Although peritoneal fluid is readily accessible during laparoscopy, analysis of its immune cells may not represent immune cell phenotypes present within endometriosis lesions. For example, tissue‐resident macrophages are known to have a different phenotype to peritoneal fluid macrophages. Peripheral blood monocyte‐derived macrophages may be plated and activated using different cytokines and estradiol to generate a phenotype similar to an endometriosis macrophage 38; this model is useful when large numbers of cells are required for gene expression and functional studies. Due to the small size of lesion biopsy material, flow cytometry for the isolation of lesion‐resident immune cells is extremely difficult and limits any manipulation of these cells ex vivo.

#### Nerve fibers

Mechsners’ group analyzed a chicken dorsal root ganglia (DRG) explant model to explore the effects of peritoneal fluid on neurite outgrowth 39. DRG are thought to contain predominantly sensory neurons, and this group were also able to recover sympathetic ganglia to aid their studies showing an imbalance between sensory and sympathetic innervation in lesions of women with endometriosis; incubation of chicken DRG with peritoneal fluid from women with endometriosis resulted in increased neurite outgrowth from sensory ganglia and a decrease in neurite outgrowth from sympathetic ganglia 40. Rat DRG explants have also been used to explore both endothelial cell–nerve and macrophage–nerve crosstalk to explore the mechanisms underlying endometriosis‐associated pain 15, 38, whereas dissociated rat DRG neurons have been used in gene expression studies 15, 38. Human embryonic stem cells can be rapidly converted into sensory neurons with a nociceptor‐like phenotype using small molecule inhibitors 41, this technique holds great potential as a platform for exploring peripheral nerve mechanisms in endometriosis. Recently, this model has been used to explore ER‐mediated expression of nociceptive ion channels 42.

#### Endothelial cells

Ectopic endometrial fragments must acquire a vasculature to survive and form a lesion; moreover, invading immune cells extravasate from the bloodstream into vascularized lesions to contribute to the inflammatory environment of the lesion, hence neovascularization/angiogenesis are an important process in the etiology of endometriosis. Anti‐angiogenic drugs are being explored as a potential therapy for endometriosis and endothelial cells are used as a model in endometriosis research. To our knowledge there are no studies documenting the isolation and propagation of endothelial cells from endometriotic lesions. Endothelial cells can be isolated from the human endometrium 43 and have been immortalized and shown to retain their phenotype in culture 44. These human endometrial endothelial cells (HEECs) have been used in endometriosis research exploring the crosstalk between endothelial cells and nerve fibers; conditioned media from rat DRG can enhance network formation by HEECs 15. Human umbilical vein endothelial cells (HUVECs) are also often used as an endothelial cell model for angiogenesis research. However, endothelial cells are known to have vascular‐bed‐specific responses and HUVECs are isolated from macrovessels compared with microvessels like the endometrial endothelial cells. To this end, HUVECs express several extracellular matrix proteins not expressed in HEECs 43.

### Three‐dimensional in vitro models in endometriosis research

Co‐culture models can be used to assess the effect of one cell type on another 45. Three‐dimensional (3D) cell culture models are reported to mirror phenotype and gene expression in vivo more closely than 2D culture systems, moreover 3D culture offers an opportunity to explore the interactions between different cells relevant to endometriosis research. Ex vivo culture of human endometrial tissue in a 3D fibrin matrix has been shown to mimic the early stages of endometriosis invasion, gland and stroma formation and sprouting of new vessels 46. Recent advances in microfabrication technologies have enabled researchers to more closely mirror physiological interactions and microenvironment of human tissue. “Organs‐on‐Chips” 47 use a microfluidic system to reconstitute the organ architecture on the chip. The major cellular constituents of the endometrium are isolated and then reassembled in microfluidic devices that are fabricated with biocompatible materials and hydrogels. These devices individually compartmentalize each cell type in independent chambers and allow imaging. Furthermore, “organ” to “organ” communication can be assessed by connecting two “organ‐on‐chips”; in the review the authors suggest that placing a “liver‐on‐chip” upstream of an “endometrium‐on‐chip” has the potential to assess the effect of metabolites on endometrial function 48. This approach could potentially revolutionize our in vitro studies and allow identification of novel targets and testing of potential therapeutics in endometriosis without the need for animal models of disease.

### Animal models

In vivo models can be used to investigate the effect of the presence of endometriosis lesions on functional outcomes such as fertility or pain that requires behavior analysis. Moreover, in vivo models allow the preclinical testing of potential therapies that are required for us to move towards anxiously awaited new therapeutic options. Spontaneous endometriosis occurs only in humans and some primates such as rhesus monkeys and baboons 49, that have menstrual cycles. Non‐human primates offer the most physiologically relevant animal model of endometriosis in terms of phylogeny and reproductive anatomy but their uses are limited due to cost and ethical concerns. The study of endometriosis in the Baboon is an attractive option for a number of reasons, including noninvasive cycle monitoring, continuous breeding, collection of naturally occurring peritoneal fluid, spontaneous retrograde menstruation and human‐like minimal to severe endometriosis. Research in baboons has been pivotal in our understanding of some key pathophysiological features of endometriosis, which have been reviewed elsewhere 50. Rodent models of endometriosis have been developed and as they offer a more tangible laboratory model of the disease they will be the focus of this section of the review.

#### Rat

The rat autologous model as developed by Vernon and Wilson in 1985 51 uses intact animals allowing the exploration of the effects of endometriosis on fertility and fecundity and has been used historically to demonstrate that ectopic endometrium is associated with an increased frequency of luteinized unruptured follicles and altered follicular development 52. This model was modified slightly by Berkley et al. 53, who sutured small pieces of uterus not only to the mesenteric cascade but also onto the abdomen and ovary to more closely mirror the distribution of lesions in women. Berkley's group was the first to use the rat model to explore the association between endometriosis and increased pelvic nociception and demonstrated that the rats had vaginal hyperalgesia in‐line with viscera–visceral referred hyperalgesia that could be suggestive of altered pain responses at the central nervous system level 53. This study preceded a number of pivotal publications that broke new ground on our understanding of pain mechanisms in endometriosis; the group were the first to demonstrate that lesions are actively innervated 54, they demonstrated that endometriosis influences pain behaviors induced by ureteral calculosis 55 again exploring the phenomenon of “viscera–visceral” hyperalgesia, and that estradiol levels modulate endometriosis‐induced vaginal nociception 56, 57, innervation, vascularization and growth factor content of lesions 58. Much of our understanding of pain mechanisms in endometriosis has stemmed from these publications.

In a refinement of the autotransplant model, MRI was used to serially and noninvasively monitor lesion growth and to obtain lesion volumes allowing for a reduction in animal usage 59. Subsequently, the rat model has been used in many studies as a test‐bed for potential therapeutics including melatonin 60, doxycycline 61, Etanercept (tumor necrosis factor α antibody) 62, and gene therapy delivered via polymeric micelles 63. The natural, mixed dopamine receptor antagonist Levo‐tetrahydropalmatine (L‐THP) was shown to reduce lesion growth and to alleviate generalized hyperalgesia 64. This list is certainly not exhaustive and we apologize to authors of the studies we cannot cite due to reference limitations. Zhao et al. also demonstrated an up‐regulation of gene expression in dorsal root ganglia that was associated with increased nociception and this was attenuated by L‐THP treatment 64. The rat model has also been used to demonstrate that tissue inhibitor of matrix metalloproteinase 1 (TIMP1) secreted from ectopic uterine tissue explants resulted in poorer embryo quality and deleterious effects on ovulation 65 and the authors suggested that novel TIMP‐1‐modulating therapies may be developed to alleviate infertility in women with endometriosis.

Recently, the rat has been used to explore pain mechanisms using innovative techniques. Dmitrieva et al. used telemetric assessment to record visceromotor responses induced by vaginal distention, allowing observer‐independent recording 66. In a unique take on the rat model, Alvarez et al. grafted autologous uterine tissue onto the gastrocnemius muscle allowing for in vivo electrophysiological recordings from sensory neurons innervating the graft and the exploration of agents injected directly into the graft 67. Uterine fragments have also been grafted onto the sciatic nerve to mimic neuropathic pain that might arise in endometriosis 68. Although these studies allow in‐depth mechanistic studies on innervating peripheral nerves, this model lacks authentic interactions between peritoneum and ectopic endometrium and does not allow physiologically relevant exploration of spinal cord engagement and vicera–visceral hyperalegsia resulting from convergent neurons. For example, using the Vernon and Wilson model, Chen et al. elegantly showed pelvic organ cross‐sensitization mediated via p38 in the rostral ventromedial medulla of the spinal cord 69.

A number of studies evaluating the validity of the rat as a model of endometriosis have analyzed the gene expression profiles of ectopic tissue deposits in the rat with the aim of comparing them to endometriosis lesions in women. Many common pathways were identified including the inflammatory response, angiogenesis, extracellular matrix re‐modeling and wound healing 70, 71, 72. Although the rat model replicates certain aspects of the disease, all of the modifications discussed above rely on the suturing of uterine fragments (endometrium plus myometrium) onto different sites and do not truly simulate dissemination of tissue into the peritoneum and formation of lesions from shed endometrial tissue. One study explored the use of fibrin glue and found less local inflammation and fewer adhesions at the site of the graft 73 but again this study used full thickness uterine fragments. An obvious limitation of using the rat model is the low number of commercially available animals engineered to have genetic manipulations. In this respect mouse models clearly have an advantage over the rat model.

#### Mouse

Since the publication of the initial autologous mouse model of endometriosis 74, which was based on the rat model described by Vernon and Wilson, many adaptations and refinements have been published (summarized in Table 2). Using mice to model endometriosis has the added benefit of the wide availability of genetically engineered animals that express ubiquitous or cell‐specific fluorescent proteins to track cells or monitor explants as well as conditional or constitutive gene deletions to aid in studies investigating gene function.

**Table 2:** Comparison of different endometriosis mouse models

Model	Benefits	Limitations
Heterologous (intact tissue)	Humanized mouse model of endometriosis. Human tissue can be manipulated before xenografting	Uses immunodeficient mice – cannot analyze full immune cell complement. Usually grafted subcutaneously – does not mirror authentic endometrium–peritoneum interactions
Heterologous (human endometriosis cell lines)	Bypasses problems in accessing tissue and variability of patient material. Used for pain studies	Immunodeficient mice. Not a true recapitulation of an endometriosis lesion
Autologous	No rejection response and good for analysis of immune cell contribution	Does not allow analysis of host/donor contribution
Syngeneic (suturing tissue to peritoneal lining)	Easy to localize lesions and measure regression in drug treatment studies. Induced in intact mice – useful for studies on fertility. Genetic manipulation of donor or host	Suturing induces an inflammatory response
Syngeneic (injection, whole uterine fragments)	Immunocompetent recipient mice. Genetic manipulation of donor or host	Uses ovariectomy and supraphysiological levels of estradiol. Difficult to localize all lesions unless reporter mice used as donors or labeling of tissue. Both myometrium and endometrium injected
Syngeneic (injection of ‘menstrual’ material)	Lesions phenocopy those recovered from women. Mirrors process of retrograde menstruation. Mice exhibit changes in sensory behavior and molecular changes in nervous system	Uses ovariectomy and supraphysiological levels of estradiol. Difficult to localize all lesions unless reporter mice used as donors or labeling of tissue

##### Human tissue xenografts

The availability of mice homozygous for the *Prkdc* ^SCID^ (severe combined immunodeficient mice) or mice with a mutation in the forkhead box protein N1 (*FOXN1)* gene (Nude mice; athymic with greatly reduced number of T cells) allows human endometrium to be xenografted either subcutaneously 75 or intraperitoneally 76 to generate humanized models of endometriosis because the mice do not mount a rejection response. This approach is very useful for determining in vivo how a “human” lesion would respond to a treatment and for elegantly determining cells originating from endometrial tissue vs. peritoneum/mesothelium 77. Because these animals are immunocompromised, injection of endometrial tissue into the peritoneum results in the formation of lesions and the tissue does not require suturing. Human endometrial tissue can also be manipulated in vitro before xenografting into the host 78. An alternative heterologous model uses human immortalized endometriosis epithelial and stromal cells that are re‐suspended in matrigel and xenografted intraperitoneally into ovariectomized, estradiol‐supplemented nude mice 79. However, lesions that form using this model cannot truly recapitulate the histological appearance of typical endometriosis lesions because they are not intact tissue fragments. These heterologous models limit the analysis of the host immune response to ectopic tissue; the inflammatory response being a key area for exploration of the pathophysiology of endometriosis.

##### Syngeneic mouse models

Syngeneic models of endometriosis bypass the limitation of the heterologous models and allow full analysis of the immune system. It is still unknown if the immunological perturbation observed in women with endometriosis is a fundamental defect of the immune system or a consequence of endometrial tissue at ectopic sites. Using a syngeneic model, induction of endometriosis was shown to inhibit spleen leucocyte function 80, which has clear implications for immunological therapies.

A number of variations of the syngeneic model exist; donor uterine tissue (full thickness or endometrial fragments only) are sutured onto the peritoneal lining or injected into the peritoneal space using a syringe or small laparotomic incision 81. Donor material is usually collected at estrous or following pregnant mare serum gonadotropin 82 or estradiol priming 83. A recently published study from our group used mouse “menstrual” donor endometrial tissue to inoculate syngeneic recipients 84. “Menstrual” endometrium was generated by hormonal manipulation using subcutaneous estradiol injections and a progesterone implant plus a decidualization stimulus to mimic the human menstrual cycle 85. Following progesterone withdrawal (4 h) decidualized bleeding endometrium was recovered and used to inoculate ovariectomized and estradiol‐supplemented recipients. Lesions were recovered after 21 days that mirrored human lesions in histological appearance, vascularization, innervation and influx of macrophages 15, 38, 84. More recently we have used our model to demonstrate that mice with endometriosis lesions exhibit robust changes in sensory behavior (both spontaneous and evoked) and elevated cyclooxygenase‐2 expression in the spinal cord and brain indicative of central sensitization (Greaves et al., 2017, accepted). The model can be used for preclinical testing of potential therapies for targeting endometriosis‐associated pain; we have demonstrated that a highly selective prostaglandin E2 receptor (EP2) antagonist could significantly attenuate both abdominal and secondary hyperalgesia.

Interestingly, the spiny mouse (*Acomys cahirinus*) was recently found to undergo spontaneous decidualization, endometrial shedding and bleeding, demonstrating for the first time menstruation in a rodent 86. Whether this mouse spontaneously develops endometriosis and its use in endometriosis research is still to be determined.

A number of studies describe an improvement on versions of the mouse model of endometriosis to allow non‐invasive monitoring of lesions. In one such study, human endometrial fragments were transduced with green fluorescent protein, transplanted into nude mice and imaged through the skin. However, because the adenoviral expression vector is transient the fluorescence faded by the third week so longitudinal studies are limited 87. Another study used donor mice engineered to ubiquitously express luciferase and uterine fragments were sutured to the peritoneal wall of non‐luminescent recipient mice. Anesthetized mice were injected with luciferin (intravenously or intraperitoneally) and bioluminescence was imaged. This approach was used successfully to monitor the efficacy of antiangiogenic therapy 88. The ability for non‐invasive monitoring of lesions is very desirable for preclinical models of endometriosis.

##### Spontaneous model of endometriosis

A spontaneous model of ovarian endometriosis has also been described with induced expression of oncogenic *K‐ras* (V‐Ki‐ras2 Kirsten rat sarcoma viral oncogene homolog) in ovarian surface epithelium resulting in benign epithelial lesions on the ovary that exhibit a simple endometrioid glandular structure; however, no associated stroma was observed 89. This activated *K‐ras* model was modified by xenografting “menstrual” endometrium expressing activated K‐ras into a subcutaneous pocket on the ventral abdomen in intact immunocompetent recipients 90.

Many of the published mouse models use ovariectomized mice supplemented with estradiol as this avoids the natural variations in estradiol in mice during the estrous cycle, and uniform availability of estradiol (usually at higher concentrations than in intact mice) is believed to promote lesion establishment and growth. This does, however, prevent the analysis of how endometriosis affects fertility. Endometriosis can be induced in intact mice, but this model uses full thickness uterine fragments sutured to the peritoneal wall. The intact model has been used to demonstrate that endometriosis is associated with decreased oocyte and embryo quality 91 and is associated with reduced pregnancy rate 92. These studies merit further exploration of the link between fertility and ectopic tissue.

## Summary

The etiology of endometriosis is complex. The availability of human tissue for endometriosis research has provided informative descriptive data on the disorder, and the adoption of internationally agreed‐on standard operating procedures for tissue and fluid sample collection (the EPHect initiative) will significantly advance this research approach. An additional layer of complexity is disease‐associated epigenetic regulation and studies should be undertaken on the epigenetic component of endometriosis and chronic pain susceptibility. Genomic approaches and next‐generation sequencing are now more affordable than ever, making these large‐scale studies more achievable. Model systems are required to explore disease‐specific mechanisms and also to validate genomic pathways discovered using GWAS and other genomic approaches. The advent and availability of different models for use in endometriosis research has considerably improved in recent years; however it is important that the research question be carefully considered before selection of the most relevant models. The physiologically relevant menstruating mouse model of endometriosis also exhibits robust changes in sensory behavior and molecular alterations in the central nervous system highlighting the model as particularly promising for the study of pain mechanisms in endometriosis, which still remain largely unknown. Reversal of the endometriosis‐associated pain state has also been demonstrated with a highly specific EP2 receptor antagonist indicating that this model is an ideal preclinical platform for testing potential therapeutics for endometriosis‐associated pain. Syngeneic mouse models of endometriosis can be used to easily explore cellular contributions of donor/host tissue and genetic manipulation can be used to enhance this. The syngeneic model also has the added benefit of a full immune system, so they can be used to explore this important aspect of endometriosis pathophysiology. Many of the published mouse models of endometriosis can be further improved by adapting them to allow non‐invasive in vivo monitoring of lesion size.

This review has highlighted the use of many different cell types and models in use for endometriosis research. Wherever possible, primary cells should be isolated from patient samples and sample sizes should be selected to achieve appropriate power. When this is not possible, models should be chosen with care. Given the heterogeneity of patient endometriosis samples, data generated using cell lines derived from endometriosis tissue must be interpreted with caution (generated from one patient). New microfluidic techniques for 3D cell culture hold particular promise for enhancing in vitro studies.

The real power of these models can be seen when ex vivo human tissue, in vitro and in vivo techniques are used in combination to produce high‐quality, clinically relevant data that advances our understanding and identifies possible future therapeutic targets.
